# Smart Supra- and Macro-Molecular Tools for Biomedical Applications

**DOI:** 10.3390/ma13153343

**Published:** 2020-07-27

**Authors:** Mariana Pinteala, Marc J. M. Abadie, Radu D. Rusu

**Affiliations:** 1 “Petru Poni” Institute of Macromolecular Chemistry, Romanian Academy, Grigore Ghica Voda Alley, 41A, 700487 Iasi, Romania; pinteala@icmpp.ro (M.P.); marc.abadie@icmpp.ro (M.J.M.A.); 2 Institute Charles Gerhardt Montpellier, Bat 15, CC 1052, University of Montpellier, 34095 Montpellier, France

**Keywords:** smart polymers, temperature-sensitive polymers, pH-responsive polymers, hydrogels, controlled drug delivery, gene therapy, non-viral vectors, bionanoconjugates, biomedical applications

## Abstract

Stimuli-responsive, “smart” polymeric materials used in the biomedical field function in a bio-mimicking manner by providing a non-linear response to triggers coming from a physiological microenvironment or other external source. They are built based on various chemical, physical, and biological tools that enable pH and/or temperature-stimulated changes in structural or physicochemical attributes, like shape, volume, solubility, supramolecular arrangement, and others. This review touches on some particular developments on the topic of stimuli-sensitive molecular tools for biomedical applications. Design and mechanistic details are provided concerning the smart synthetic instruments that are employed to prepare supra- and macro-molecular architectures with specific responses to external stimuli. Five major themes are approached: (i) temperature- and pH-responsive systems for controlled drug delivery; (ii) glycodynameric hydrogels for drug delivery; (iii) polymeric non-viral vectors for gene delivery; (iv) metallic nanoconjugates for biomedical applications; and, (v) smart organic tools for biomedical imaging.

## 1. Introduction

The exponential evolution of macromolecular science reached the natural point in which polymeric materials are required to display a “smart” functional character. To sustain life and perpetuate biological tasks, nature uses dynamic transformations of particular (macro)molecular edifices and interfaces with selective structures and functions that respond to their surroundings. Inspired by the ancestral need to mimic nature, the progress of stimuli-responsive polymers is based on the quest for similar stimuli-sensitive building blocks, architectures, and mechanisms in order to reach biological intelligence in a less intricate fashion [1,2,3]. These macromolecular constructs are able to modify their shape, volume, solubility, supramolecular arrangement, and other structural or physicochemical attributes in response to an environmental trigger [4,5,6].

Accordingly, biomaterials with finely tuned, smart functions, and spatiotemporal control are at the heart of biomedical research and they represent a key step in the evolution of patient-centered care [7]. The topic’s complexity is overwhelming, but the common, key element of the smart-like demeanor is a non-linear response to a physiological microenvironment (pH, redox processes, enzymes, glucose) or an exogenous factor (e.g., temperature, light, solvent nature). There is a wide range of chemical, physical, and biological motifs available for the fabrication and engineering of smart biomaterials.

Nevertheless, most of the research endeavors that are dedicated to the subject are based on the individual or cooperative responses to two major triggers: temperature and pH. In a similar fashion to biological processes, the overall smart reaction is the sum of various synergetic interactions, like H-bonding alteration, electrostatic changes, or ionization. Taken one by one, these changes have a subtle, low energetic nature, but they add up along the macromolecular structure into solid modifications and biologically-relevant responses [8].

This short review compiles (among others) some of the most interesting results recently obtained (in the last five years) by our group in the broad field of smart supra- and macro-molecular tools for biomedical applications. The classical building blocks, already well-established architectures and major mechanistic details that are involved by these topics are already approached by many valuable systematic literature reviews [1,2,3,4,5,6,7,8], of both narrative and best-evidence methodologies, and it is not our wish to reproduce or simply upgrade them. The goal of this work is to evaluate the status quo, as evidenced by expert opinions in the field, to narrow the topic, and to isolate and underline the impact of these tools on the resulting architectures and following bio-related potential. Although our research spans diverse topics, from bioagents’ carriers, biosynthesis [9,10,11], genotypic mapping [12,13,14,15,16], and cell growth [17,18], to artificial water channels [19,20,21,22], and the exploration of investigative methods [23,24], the review considers the hot topics of drug and gene delivery, antioxidants development, and bioimaging. We addressed these topics by following five major themes: (i) temperature- and pH-responsive systems; (ii) glycodynameric frameworks; (iii) polymeric non-viral vectors; (iv) metallic nanoconjugates; and, (v) smart organic tools. We condense and direct our speech towards insufficiently explored, smart synthetic instruments, design and mechanistic details, subsequent architectures, and particular insights that led to stimuli-responsiveness or to biomedical promises. These represent additional starting points or refined research pathways in the quest for smart solutions for the ever-demanding challenges of the biomedical realm.

## 2. Temperature- and pH-Responsive Systems for Controlled Drug Delivery

The development of drug therapy under controlled or retarded delivery conditions is a major achievement of the academic and industrial research society. Several pharmaceutical formulations have made it into the market after proving able to afford a constant drug concentration in the biological environment from a single administration. This translates into a decreased dose frequency and enhanced therapeutic efficiency, patient comfort, and compliance [25,26,27,28].

Despite its obvious benefits, the controlled drug delivery strategy shows some limitations, being neither sufficient nor effective in clinical circumstances in which the bioactive species should be released only when the physiological parameters are altered. These include diabetes, angina pectoris, heart rhythm disorders, etc. and they require tailored formulations entitled self-regulated drug delivery systems, usually based on stimuli-sensitive polymers [29].

From the broad variety of available materials, temperature-responsive polymers are the preferred systems since they operate based on changes in the human body temperature to amend innate features and trigger drug release [30,31]. Poly(N-isopropylacrylamide) (poly(NIPAAm), one judicious abbreviation of many available) has evolved as the classic choice between thermosensitive polymeric systems. Its popularity comes from a phase transition in aqueous solutions, which occurs at temperatures (lower critical solution temperature, or LCST) close to the human body, at 32–34 °C [32], due to a fast change of hydrophilicity and hydrophobicity along the polymeric chain. Below LCST, the system is highly hydrated and the linear polymer is soluble in water or the cross-linked homolog swells. Above LCST, the hydration decreases and the polymer precipitates, while the corresponding network collapses.

The microporous, three-dimensional architecture of hydrogels is usually favored over linear systems, since it empowers a set of particular, versatile physicochemical features, which unlocks a plethora of biomedical possibilities, especially supporting drug entrapment and its controlled release [33,34,35,36,37]. The volume changes determined by an ideally fast swelling and deswelling of the three-dimensional (3D), soft network are at the heart of drug delivery at a directed pace [32,38]. They are governed by the diffusion of water throughout the cross-linked matrix, which is further boosted by small-sized hydrogels [39,40].

One drawback concerning poly(NIPAAm) is a decrease in the LCST value when placed in a salt-rich, physiological environment with high ionic strength [41]. Therefore, poly(NIPAAm)-based systems with LCST close to the human body temperature and a sharp phase transition in a narrow temperature range are key requirements for optimized controlled drug delivery and they represent the core of an extensive research topic in the field.

Generally, this goal is achieved by copolymerizing NIPAAm with hydrophilic monomers [42,43]. The comonomer must be judiciously chosen, since alteration in the poly(NIPAAm) structure usually results in a modification of the hydrophilic/hydrophobic equipoise, hydrophobic interactions at temperatures above LCST, and an undesired decline of the overall thermal responsiveness.

A viable option in this regard is N-isopropylmethacrylamide (NIPMAAm), a comonomer that is quite close to NIPAAm in structural terms, especially when it comes to the succession of amide and isopropyl functionalities. While being more hydrophobic than the classic system, poly(NIPMAAm) contains methyl units along the backbone that lessen hydrophobic interactions and lead to a sharp phase transition at higher LCST, around 46 °C [44]. When copolymerized in a 51:49 co-monomer ratio, a thermosensitive poly(NIPAAm-*co*-NIPMAAm) system with an LCST value (36.8 °C, determined in simulated physiological conditions: PBS, pH 7.4) close to the human body temperature is generated [29]. Moreover, the similar chemical nature of the building blocks allows the much desired sharp phase transition of the resulting system. The copolymer is further cross-linked with N, N′-methylene bisacrylamide (MBAAm) in a water:methanol mixture. The obtained thermoresponsive microgels undergo swelling and deswelling at quite a high pace, below and above a VPTT (volume phase transition temperature, a more appropriate term than LCST for cross-linked systems) value of 33.7 °C. The difference between the critical temperatures comes from two synergic processes. On one side, the macromolecular chains’ flexibility is hampered by the cross-links, and hydrophobic interactions will require a different, usually higher energy (and subsequent temperature). On the other, low cross-linked networks can be regarded as concentrated polymeric solutions of lower LCST values, in opposition to the diluted solutions for which the normal LCST is determined [45].

Drug delivery vehicles are prepared afterward by loading a model drug (dexamethasone) into the microgels via the solvent evaporation method, which enables a progressive uptake of the drug within the polymer network until drying. Cyclical temperature changes below (32 °C) and above (38 °C) VPTT generated a drug release mechanism (in simulated physiological conditions) of a pulsatile fashion in the cross-linked microgels.

Interesting results can be obtained when choosing comonomers of a more different chemical nature. For example, the copolymerization of NIPAAm with N-vinylpyrrolidone (NVP) in a 91.5:8.5 ratio affords thermoresponsive systems with an LCST value of 36 °C in simulated physiological conditions [46]. Because the two comonomers have weak ionizable units, the LCST is sensible to the surrounding ionic strength and pH, higher LCST values (37.3 °C) being obtained in a simulated gastric fluid environment (pH 1.2). Further studies on copolymer solutions with various salts show a general decrease in the LCST values with the increase of the ionic strength. One riveting feature is the strong influence of the ion size on LCST values. While the critical temperature is indifferent to the change between Na^+^ and K^+^ cations, the substitution of Cl^−^ with H_2_PO_4_^−^ or CH_3_COO^−^ generates drastic changes of the LCST values.

Two-phase suspension polymerization (under nitrogen) of the same comonomer ratio was used in order to synthesize cross-linked microspheres (the same MBAAm cross-linker as above). Diclofenac was loaded (via physical interactions) as small crystalline microdomains in these microspheres by solvent evaporation. The investigation of the release mechanism revealed a strong dependency on the percentage of the loaded drug. Low-loading microspheres (7.62% diclofenac, *w*/*w*) provide a pulsatile release mechanism under cyclical variations of the temperature below and above VPTT. High-loading microspheres (13.08% diclofenac, *w*/*w*) display a much slower release rate, due to a sharp reduction of the temperature effect and collapse of microspheres determined by the abundance of drug crystals on the microspheres’ surface and by the matrix’ lowered flexibility, respectively.

The “smart” character of poly(NIPAAm) systems can be further tailored to add a second trigger, pH, to their stimuli responsiveness. This will enhance their drug delivery capacity in physiological environments of differing pH, like the gastrointestinal tract. The trigger impacts weakly acidic or basic structural motifs within a (co)polymeric system, while its swelling and collapsing come from the protonation and subsequent deprotonation of these pH-sensitive functionalities [47,48]. The problematic part of this pathway is a severe reduction or even revocation of the thermoresponsive character due to the high hydrophilicity of the ionized pH-sensitive comonomer and the alteration of the overall hydrophilic/hydrophobic balance of poly(NIPAAm) [49,50]. The charged pH-sensitive molecules are prone to electrostatic interactions with hydrophobic units of opposite charge (coming from a drug, for example) and the thermally triggered sensibility can be restored. This is the major design framework that is involved in the efficiency of dual-responsive systems.

**Figure 1:**
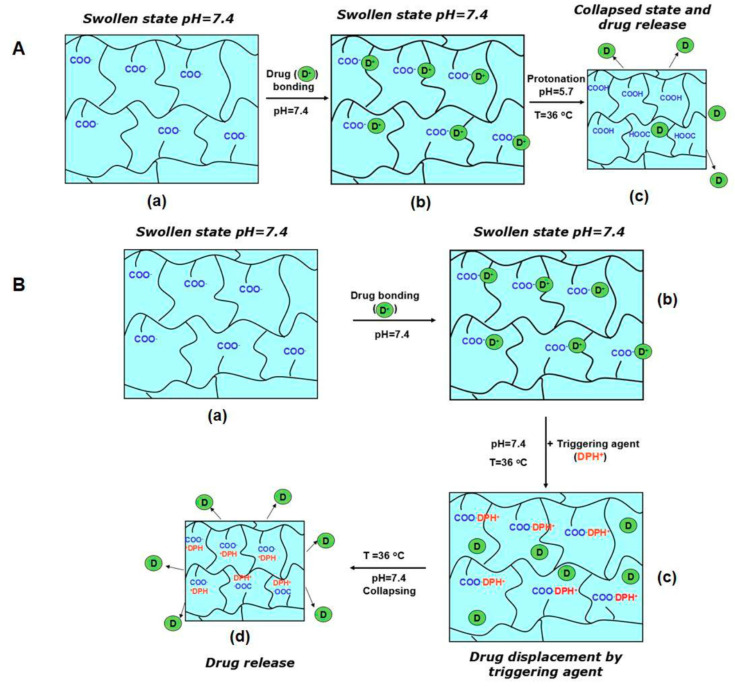
Schematic representation of the operating principle of pH- and thermo-sensitive poly(NIPAAm-*co*-MAc) microgels in simulated physiological fluids. (**A**). isotonic solution, pH 5.7; (**B**). PBS, pH 7.4: (a) swollen microgels in carboxylate form; (b) drug loading via electrostatic interactions; (c) and (d) activation of microgles, collapsing and drug release. Reproduced from [51], with permission from © 2017 Elsevier.

Such a system is obtained by combining NIPAAm with maleic acid (MAc) in different molar ratios. The synthesis involves the use of MAc in its protonated form to obtain statistical copolymers, while the employment of a fully ionized comonomer version only leads to NIPAAM homopolymers [51]. The poly(NIPAAm-*co*-MAc) copolymer displays LCST values that are close to the human body temperature in water, acidic, and isotonic solutions. No LCST is observed in simulated physiological conditions (pH 7.4; 36 °C) due to the ionization of the hydrophilic MAc. The thermal sensitivity and drug delivery ability are recovered at physiological pH and temperature once the carboxylic MAc moieties electrostatically interact with a positively-charged, hydrophobic, bioactive molecule (e.g., diphenhydramine) (Figure 1).

Therefore, this strategy supplies copolymers with biosensory (from the carboxylic units) and delivery (from the thermoresponsive moieties) features. The corresponding thermally sensitive hydrogels follow the same behavior, acting as actuators and collapsing and, therefore, releasing the loaded drug under the influence of the external bioactive trigger.

Similar functions can be attained by replacing the maleic acid comonomer with N-(3-aminopropyl)methacrylamide (APM) [52]. Microspheric gels (glutaraldehyde as a cross-linker) that are based on poly(NIPAAm-*co*-APM) proved inactive under simulated physiological conditions (pH = 7.4 and T = 36 °C) due to the highly hydrophilic APM ionized state. Its thermal sensitivity is activated by the presence of the hydrophobic indomethacin bioactive component, and the microgels shrink and mechanically liberate the encapsulated drug. For this ternary system, the biosensor function belongs to the pH-responsive amino groups, the triggering agent is indomethacin, and the thermoresponsive NIPAAm-based network maintains its actuator (delivery) role. Moreover, the microgels prove completely non-toxic, easing a rich adherence of rabbit dermal fibroblasts to their surface.

Another approach to specifically tailor the drug delivery capability of thermoresponsive systems is to modulate their size and shape. Nanoscale polymeric micelles are an emerging trend in drug delivery, the self-assembled core-shell morphology proving an efficient pathway to load hydrophobic or poorly water-soluble bioactive molecules via hydrophobic, H-bonding, or electrostatic interactions [52,53,54,55].

Poly(NIPAAm) was used as the thermoresponsive component in various combinations in amphiphilic block or double-hydrophilic graft copolymers in order to obtain stimuli-responsive polymeric micelles with drug delivery features [56,57,58,59]. A recent, popular direction in this area is grafting poly(NIPAAm) onto polysaccharide backbones to profit from the innate abundance in structural functionalities and excellent biodegradability/biocompatibility of the latter [60]. The H-bonding between the polymeric building blocks and a hydrophobic drug allows for the formation of nanosized constructs below LCST. For example, double hydrophilic copolymers which form uniform, spherical aggregates within 40–80 nm are obtained by grafting NIPAAm on the backbone of pullulan under homogeneous conditions (below the former’s LCST) [61].

Indomethacin-loaded nanoparticles that are based on them can be obtained by dialysis and nanoprecipitation to achieve increased entrapment efficiency (up to 80%). The mean size and polydispersity of these nanoaggregates depend on the amount of loaded drug, copolymer concentration, and molecular weight of the thermosensitive block. Thermally-triggered in vitro release of the model drug is conditioned by the indomethacin content and the same molecular weight of the NIPAAm fragment.

**Figure 2:**
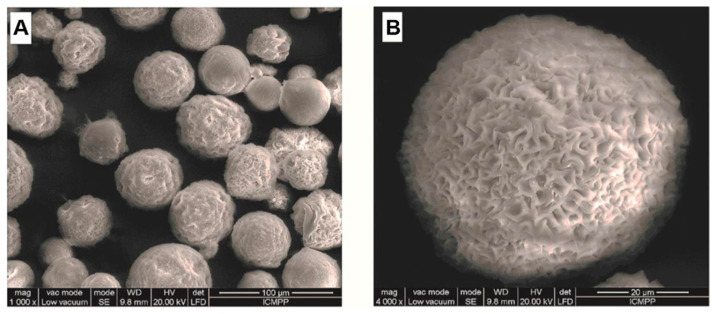
Scanning electron micrographs of dried chitosan microspheres: (**A**). general view, (**B**). surface detail. Reproduced from [62], with permission from © 2016 Elsevier.

A further step in the complexity of such systems is achieved by physically embedding drug-loaded chitosan microspheres within NIPAAm-based hydrogels [62]. Biodegradable chitosan microspheres display a very interesting, fine morphology and they are able to electrostatically load opposite-charged bioactive molecules, like salicylic acid (Figure 2).

Under physiological conditions, the competitive ions trigger a fast-displacement and release of the entrapped drug. This inconvenient release rate is tailored by the physical incorporation of chitosan microspheres into a hydrogel based on cross-linked poly(N-isopropylacrylamide-*co*-hydroxyethylacrylamide). The resulting smart composite hydrogels show a release profile, depending on both pH and temperature, while the thermosensitive matrix provides mild protection against the in vitro degradation of the polysaccharide microspheres.

## 3. Glycodynameric Gels for Drug Delivery

The use of naturally derived building blocks, like polysaccharides in the development of hydrogels, provides enhanced biocompatibility and biodegradability and it ensures a resemblance to the external extracellular matrix in terms of composition and mechanical properties [34]. Therefore, chitosan, the second most abundant natural polymer, qualifies as an excellent candidate for such biobased, soft, 3D materials, and brings to the field some valuable, innate characteristics: stability, nontoxicity, enzymatic degradation, abundant therapeutic activity, and others [63,64].

One cutting-edge direction in the evolution of hydrogels is based on the passage from the static, classical design to a more dynamic blueprint that is related to supramolecular chemistry. Dynamic (hydro)gels can be accessed through reversible cross-linking processes of both chemical or physical essence and represent a new class of smart materials with by-design or on-demand response to natural or artificial triggers [65,66,67,68,69]. The imine structural motifs (Schiff bases or azomethines), coming from the condensation of amino and carbonyl units are one of the most potent tools of the covalent dynamic strategy, an elegant entry point towards highly complex, tunable materials with dynamic architectures [70,71]. The reversibility of imines is a feature of several biological and pharmaceutical processes and it follows a fast pace dictated by intrinsic reactivity, water, temperature, or pH [72,73,74].

This multifaceted synthetic tool can be used in the generation of dynamic chitosan gels by using the abundant amine functionalities of the polysaccharide in combination with small-sized aldehydes. Such hydrogels are usually built by using glutaraldehyde and they represent biodegradable, pH-responsive materials with certain applicative possibilities [75]. However, this dialdehyde, like most of its kind, displays intolerable toxicity, which hampers its biomedical usage and it enables the quest for other building blocks with a positive impact in the biomedical realm [76,77].

One recent, viable solution to this issue is the employment of monoaldehydes in condensing the chitosan polyamine towards imine moieties [78,79,80]. At this point, dynamic chemistry boosts imination and transimination reactions (required to attain the most stable structures) and it enables self-ordering into clusters, which will further act as cross-linkers for dynamic chitosan reticulation [70,81]. Both challenging and promising, the method eludes toxic dialdehydes and uses the access to a wide range of safer monocarbonylic counterparts to unfold chitosan gels with biomedical usage.

This strategy was first implemented by using 2-formylphenylboronic acid to develop dual cross-linked supramolecular chitosan hydrogels via covalent imine connections and physical, H-bonding interactions that are based on the boronic OH groups. The low molecular weight building block shows low toxicity and a two-fold biological activity: an antifungal and anticancer character mainly coming from the boronic residue and specific bio-targeting of lipids, proteins, and cancer cells from the overall iminic framework [82,83,84]. Moreover, it offers the possibility to attain versatile morphologies and surface properties and stabilize the imine moieties through the formation of iminoboronate units [85].

Several combinations between the amine and aldehyde functionalities led to the formation of chemo-physical chitosan networks with three-dimensional order dictated by the ratio between the chemical and physical gelling [78]. Intra- and inter-molecular H bonds stabilize the imine framework and constrain the coiled chitosan backbones towards a straight conformation, thus leading to a nanostructuring that impacts the morphology at the micrometer scale and it enhances the mechanical features of the material. The interplay between the hydrophilic chitosan and hydrophobic iminoboronate generates a highly ordered, segregated, supramolecular architecture in the chitosan networks with a dominant covalent cross-linking. This affords strong, elastic materials with a high recovery degree (90%) and resistance to deformation, as compared to the less ordered hydrogels that are mostly based on physical cross-linking. As expected, they also displayed a strong antifungal activity against both planktonic and biofilm Candida yeasts, even at low concentrations (0.142%) of the boronic acid. When corroborated with a pH-tailorable swelling degree and enhanced mechanical properties, this feature qualifies the chemo-physical chitosan hydrogels for advanced investigations that are related to the treatment of recurrent vulvovaginitis infections.

**Figure 3:**
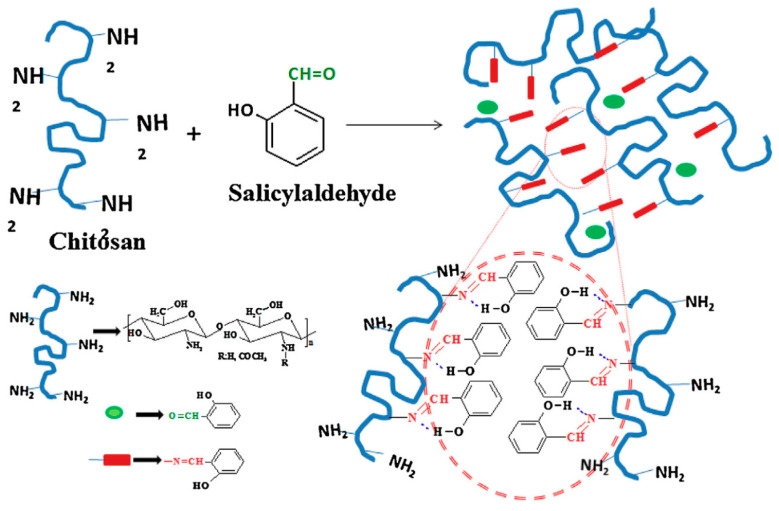
Schematic representation of the formation of salicyl-imine-chitosan hydrogel. Reproduced from [79], with permission from © 2017 Elsevier.

These initial auspicious results encouraged the further exploration of the monoaldehyde route in the construction of chitosan gels with enhanced characteristics. A naturally available monoaldehyde, salicylaldehyde, was chosen as the cross-linking agent due to its rich biological activity (antifungal, antimicrobial, anti-mycotoxigenic, chemosensing) and the FDA (US Food and Drug Administration)-approved seal [86,87,88]. The salicylaldehyde-empowered chitosan gelling was solved through a complex investigative approach, combining NMR (nuclear magnetic resonance spectroscopy), FTIR (Fourier-transform infrared spectroscopy), XRD (X-ray diffraction analysis), and rheological measurements [79]. The dynamic cross-linking was decoded as the supramolecular combination of three distinct processes: self-assembly, self-organization, and segregation. The self-assembly between the polysaccharide and monoaldehyde occurs via imine moieties that self-organize in hydrophobic associations. These are sustained by the aldehyde motion between amine functionalities in the reversible, covalent imine formation in a so-called “imine clip” effect. The hydrophobic frameworks participate in a hydrophilic/hydrophobic segregation and determine ordered clusters that operate as net nodes between the chitosan backbones (Figure 3). A minimum amine to aldehyde ratio of 3.2 was determined as being essential in the formation of chitosan cross-links by rheological measurements.

A distinct combination of mechanical and thermal features comes from this supramolecular ordering. The elastic salicyl-imine-chitosan hydrogels prove thixotropic and thermosensitive behaviors and a swelling manner that can be tailored though the cross-linking degree. Even more interesting, the particular chemical platform of these materials enables luminescent and self-healing conducts at room and body temperature, which are promising features for bio-related usage.

One appealing route of bio-application for this type of glycodynameric gels is related to precision, local medicine, and cancer targeting in particular. Multifunctional, responsive hydrogels with accessible formulation and controlled gelling time are primary candidates for injection at the tumor site if able to retain local spreading and acceptable toxicity [89,90]. In this context, the dynamic monoaldehyde strategy was used to build soft, 3D networks based on chitosan and nitrosalicylaldehyde for local tumor targeting [91]. Chitosan has an antitumor effect and its gelling takes place at basic pH, thus suiting the limited spreading of its hydrogels in the basic pH of the normal tissue, or of its precursors in infected tissues with local acidosis [92,93]. On the other side, nitrosalicylaldehyde and imines therefrom also display improved antitumor activity and bypass the toxicity issue of the dialdehydes usually employed in the development of hydrogels [94,95]. However, basic requirements like controlled gelation and acceptable toxicity need to be met first.

**Figure 4:**
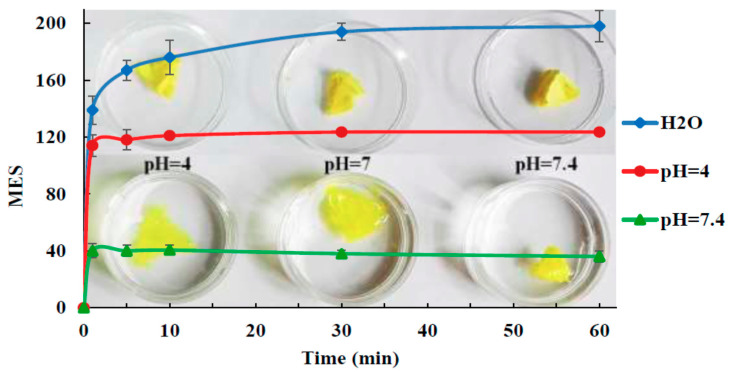
Time-dependent swelling of nitrosalicylaldehyde-based chitosan hydrogels in different pH media and their images before and after swelling. Reproduced from [91], with permission from © 2017 Elsevier.

An accelerated hydrogelation was observed under physiological pH (PBS, 37 °C) while maintaining their original shape and dimensions. In an acidic, tumor-characteristic pH (citrate buffer), the gelation occurred at a slow pace and a self-healing conduct was observed (Figure 4).

The incubation of HeLa cells proved an in vitro cytotoxicity around 60% for these superporous networks, while an excellent in vivo biocompatibility was confirmed by subcutaneous administration in rats. The success of the proof of concept confirms their potential in local antitumor therapy and certifies the feasibility of the glycodynameric pathway for bio-related applications.

The advantages of this system were even further tested by encapsulating a model drug, diclofenac sodium salt (DCF), in nitrosalicylaldehyde-imine-chitosan hydrogels as drug delivery vehicles for local therapy [96]. A careful formulation and variation of the crosslinking density enabled the anchoring of DCF into the porous matrix as sub-micrometric crystals or as isolated molecules via physical forces. In vitro release kinetics showed prolonged drug liberation in simulated physiological conditions, an operative therapeutic concentration being attained in less than one day, followed by an eight day sustained release. The therapeutic effect was effective for five days when tested in vivo by the somatic pain model on rats, an encouraging result when compared to the one-day efficiency of systemic administration [97]. The best results were obtained for the hydrogels in which DCF was finely dispersed mostly as isolated molecules, due to a slowed dissolution and diffusion. The resulting systems proved in vivo biocompatibility and biodegradation triggered by tissue-characteristic enzymes. Therefore, a synergic effect is expected for local delivery in tumors, since the chitosan matrix, its oligomers coming from biodegradation and the encapsulated active principle come with a certified antitumor effect.

A logistic model of the dynamics of these controlled drug delivery systems was developed based on the use of multifractality in logistic type laws. Triggered by the multifractal nature of drug release curves (continuous curves with varying degrees of non-differentiability), the logistic type laws were developed as a more appropriate theoretical model (a non-linear, non-differential and non-integrative approach) to replace or complement the standard, empirical mathematical method (a differential and integral approach, which suffers in describing the dynamics of drug release) in order to depict the dynamics of controlled drug release in a more pragmatic fashion. The drug-loaded polymer matrix represents a highly complex system from both structural and functional points of view: a very large number of non-linear interactions between structural elements under the constraints that were imposed by the biological environment. Such a theoretical method allows for deconstructing and simplifying the release of active principle from this intricate polymer-drug system at various resolution scales [98].

The correlation between the mathematical model and experimental data was confirmed by the linear dependence of the amount of released drug as a function of time. This theoretical strategy enables the switch from the empirical, rather chaotic description of release dynamics through the various classical models to more practical, non-linear ones that can encompass the dynamicity of the system. At the same time, it validates the assumption that complex polymer-drug frameworks obtain memory, and swelling, drug diffusion, and matrix degradation represent dynamic processes of pseudo-intelligent systems. Therefore, a mathematical confirmation of the much desired “smart” functional character is obtained.

These theoretical and experimental findings support intensive research on the topic and the exploration of additional formulations and new monoaldehydes for biocompatible, bioactive, and biodegradable drug delivery matrices for local chemotherapy.

One direction of development is the exploration of morphology control and multiplication, so as to address new bio-related applications. For example, the use of another natural, aromatic monoaldehyde, namely cinnamaldehyde, as a chitosan cross-linker in different ratios enables porous architectures of variable morphologies [99].

These can be further tuned by the interplay between chitosan’s molecular weight, cross-linking degree, and incubation conditions (time or media), leading to several pathways of fine morphological control to satisfy a targeted biomedical usage.

A broader extension of the dynamic imine-based strategy involves reaching natural monoaldehydes of a different nature, like the non-toxic, all-aliphatic citral in building chitosan-derived hydrogels. The same molecular and supramolecular dynamic features are used to obtain amphiphilic glycodynamers that reversibly immobilize the hydrophilic citral in the hydrophobic chitosan matrix by physical and covalent means [80]. The resulting ordered networks show a sponge-like microstructure, are highly elastic, and they display thixotropic and thermosensitive features similar to their homologs based on aromatic monoaldehydes, convenient traits in biomedical terms. The new gelator renders hydrogels with in vivo biocompatibility, as monitored on laboratory mice.

**Figure 5:**
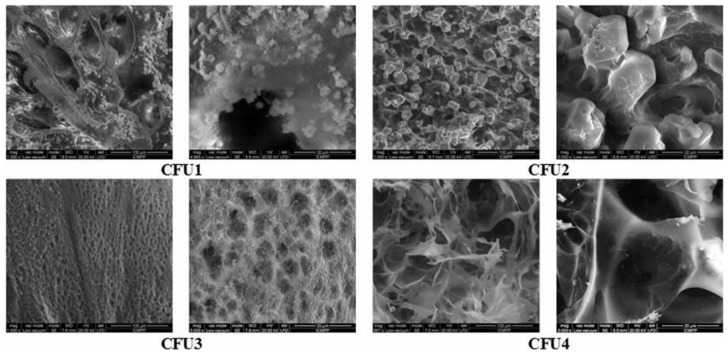
Scanning electron micrographs of citral-imino-chitosan hydrogels loaded with 5-fluorouracil. Reproduced from [100], with permission from © 2018 Taylor & Francis Group, under CC BY 4.0.

The homogeneous, microporous morphology of these hydrogels was exerted in the immobilization (during hydrogelation) of an antineoplastic drug, 5-fluorouracil, to prepare anticancer drug delivery systems [100]. The dynamic nature of these materials allowed for a uniform dispersion of the active principle of various sizes within the cross-linked matrix, while the reduced dimensional polydispersity of the network’s pores determined a homogeneous response in a biological environment (Figure 5).

Moreover, the innate thixotropy of the gels permits their targeted injection and sets the bases for a prolonged local release at the injection site (in this case, the peritoneal cavity), which assures the much-desired combination of maximum local effect and minimum overall side effects. The drug release behavior, as followed by UV–Vis and various theoretical mathematic models, consists of a prolonged, diffusion-controlled mechanism starting from the outer layers of the hydrogels [101]. The system with the lowest citral concentration acted as the best drug reservoir, releasing roughly 65% of the anticancer reagent in 32 h. The chitosan-based matrices showed in vivo side effects that were comparable and most likely belonging to the pure drug and a lysozyme-triggered enzymatic degradation (11% mass loss after 21 days). These results boost further investigation of such dynamic delivery systems in intraperitoneal chemotherapy and their extension towards the local delivery of other antineoplastic agents.

## 4. Polymeric Non-Viral Vectors for Gene Delivery

Gene therapy is a pioneering procedure that is at the forefront of medical research that employs nucleic acids as drugs in addressing pathological cells to substitute a defective gene, in order to correct a genetic imperfection, or to amend a chronic disease [102,103]. It is basically about the optimal loading, protection, and transfer of genetic material to a specific site via stable, non-toxic, biodegradable carriers or vectors that are able to cross some sophisticated and sensitive physiological barriers (e.g., cell membranes) [104,105].

While initial endeavors in the field were mostly dedicated to viral vectors (e.g., adeno-, lenti- and retro-viruses), several safety- and production-related issues moved the attention towards alternative, non-viral systems (e.g., cationic lipids, polymers, dendrimers) [106,107].

The crucial DNA-complexation skills of such a carrier must accomplish two conflicting functions that are accessible through smart polymeric tools: (i) a strong connection to the anionic, semirigid DNA chains to ensure safe passage and barrier penetration and (ii) a reversible interaction with the DNA fragments to afford proper release (in the cell nucleus) [108]. Such functional aggregates are dynamic (bio)conjugates that encompass a minimum number of molecular motifs to satisfy the required polyvalent biological tasks. They operate based on the mutual supramolecular affinity and dynamical steric complementary between the inactive (vector) and active (nucleic segment) complements [109].

The intricate transport mechanism is not completely understood, and it is customarily narrowed down to a restrained collection of computational and experimental parameters [110]. The complexity of this puzzle is further enhanced by a wide variety of vector architectures and, thus, the ideal, both efficient (transport and safety) and easy-to-manufacture non-viral carrier cannot be fully described and designed.

A potential silver lining in non-viral vectors research is the use of polycationic macromolecular segments that efficiently complex nucleic acids and gene plasmids into polyplexes while maintaining a good barrier penetration yield [111,112]. Some of the best players in the field, judging by the number of available studies, are linear or branched poly(L-Lysine) (PLL) and poly (ethylene imine) (PEI) [113,114].

Theoretical molecular dynamics simulations and experimental studies explain their success by the variable ability to neutralize the negative charges of DNA through pH-dependent, electrostatic binding and high density of amine functionalities susceptible to protonation in endolysosomal-specific pH conditions (pH 5.0–7.4) [105,110,115,116].

Computational experiments show that the binding rate of the two involved macromolecules (DNA and polycation) varies as a function of the polycationic protonation. For example, at full PLL protonation (which takes place at pH 5.4), the distance between macromolecules drops in a couple of nanoseconds (from roughly 40 Å to below 20 Å). At a smaller degree of protonation (50%, attainable at pH 7.4), the same distance between macromolecules is reached after roughly 30 s [110].

The same in silico experiments confirm that hydrogen interactions are mostly instituted between the oxygen atoms of the DNA backbone and the hydrogen ones belonging to the PLL’s amine functionalities and are stronger at lower pH (5.4, as compared to pH 7.4). Moreover, the flexibility of the two macromolecules, another important aspect in polyplex formation, is pH-dependent. While DNA has a relatively stable conformation inside the polyplex at physiological pH, PLL chains (150–300 kDa) prove more flexible and it is prone to bending and twisting in these conditions.

PEI has a higher concentration of amines that generates a pH buffering ability and contributes to an efficient endosomal escape of polyplexes via a so-called ‘proton-sponge’ mechanism [105]. Moreover, it assembles DNA in polyplexes that are stable in physiological conditions while being prone to replisomes-triggered degradation. Therefore, cationic, water-soluble, linear, and branched PEI frameworks are considered by many the gold standard in DNA and RNA delivery, both in vitro and in vivo [117,118]. The major drive in the PEI-based vectors research is the quest for additional building blocks that allow modified PEI architectures with enhanced transfection and low cytotoxicity. The studies usually express DNA binding efficiency in relationship with some design and experimental elements: the ratio between the carrier’s elements, their size (especially PEI’s) and electrical charge, overall cytotoxicity, and N/P ratio (N: nitrogen content from the polymeric unit, P: phosphate content from DNA).

The aforementioned supramolecular dynamic strategy was used by our group to develop a synthetic platform for the creation of libraries of modular carrier frameworks that self-adapt in a dynamic fashion to the genetic material target [119,120,121,122]. The self-adjustment is established on reversible interactions (imine units, hydrophilic/hydrophobic combinations, H-bonding, electrostatic interplays) between the frameworks’ elements and the target or biological environment, which grant the optimal modulation of 3D geometry and functional characteristics. Besides, the dynamic strategy affords the full screening of a broad range of combinations and a fast selection of the most effective carriers.

**Figure 6:**
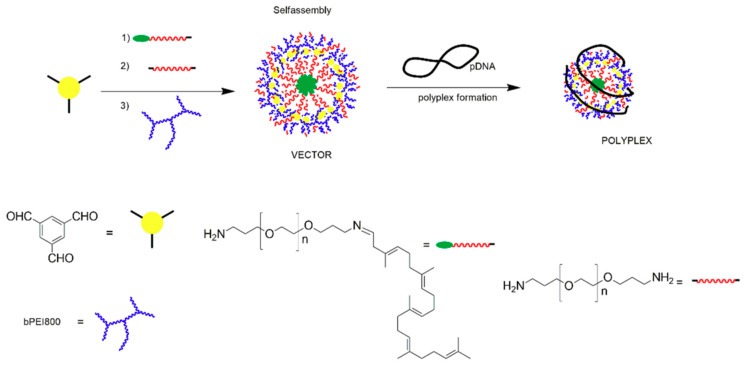
Schematic representation for the formation of dynamic multi-component vectors and corresponding polyplexes. Reproduced from [122], with permission from © 2019 MDPI, Basel, under CC BY 4.0.

The libraries were constructed, starting from a trifunctional core (benzene-1,3,5-tricarbaldehyde) that reversibly connects the frameworks’ components and DNA-complexation sites into hyperbranched structures by using amino-carbonyl/imine synthetic tools (Figure 6) [122].

Three types of components are used in various ratios: (i) low molar mass, branched PEIs as the DNA-binding cationic units; (ii) amine-terminated PEG (polyethylene glycol) chains of various sizes as the hydrophilic component and immunogenicity-reducing agent; and, (iii) PEGylated squalene as the hydrophobic element. Small-sized, branched PEI segments were chosen, since preliminary studies showed that higher molar masses promote undesired cytotoxicity [123,124]. PEGylation was applied to increase cargo flexibility and solubility, and to shield the polyplex surface charge by a so-called “stealth effect”, which however might decrease transfection yield by reducing ionic interactions with target cells [125,126]. The unsaturated lipid derivative was introduced based on its high biocompatibility and the ability to self-assemble as to minimize the contact with aqueous environments [127].

The first set of structure-performance studies showed a cooperative impact of the low PEI (2 kDa) and PEG (1.5 kDa) moieties within the dynamic frameworks on promoting high transfection efficiency whilst reducing the toxicity against human cells [120]. Squalene assisted the self-assembly of cargo frameworks into supramolecular amphiphilic architectures that form stable polyplexes with DNA and safely deliver it to cells. The carriers’ DNA-binding ability was directly connected to the PEI concentration at low N/P values, while the amine-decorated PEG enhanced the transfection at higher N/Ps. The library-based assessment also determined the most favorable vector composition in terms of PEI concentration (1.5 equiv.), aminated PEG content (1 equiv.), and N/P ratio (50) in order to reach the highest DNA transfection to HeLa cells. The best performing polyplexes displayed suitable dimensions for cellular uptake and appropriate cytotoxicity.

A consecutive study used smaller, branched PEI fragments (0.8 kDa) and three PEG segments of different sizes (1.5, 2.0, and 3 kDa) within the same supramolecular architecture, as above. The collection of non-viral carriers was engineered this time to follow the effect of the systematical increase in the ratio and size of the amine-terminated hydrophilic units on the dimensions, DNA-binding, and subsequent transfection and toxicity of the resulting vectors [122].

A size-dependent, sterical interplay between the PEG segments and other components was observed, which also affected the DNA-complexation features. The longer PEG chains generated smaller particles and reduced DNA-binding, which resulted in larger and loosened polyplexes, due to a shielding effect over the branched PEI segments. At the same time, they afforded the carriers with the highest biocompatibility, but lowered the in vitro transfection efficiency. Optimal, 1 equiv. ratio of aminated PEG and N/P ratio of 100 were found to provide the best transfection performance.

Morphological evaluation of some of these carrier systems substantiated high discrepancies in the dimensions of PEG-based systems as compared to their non-PEG counterparts. The absence of PEG chains determines an intricate cross-linking process, followed by a PEG-driven self-aggregation into even bigger assemblies.

Nanospherical carriers can be obtained through the reversible imine strategy by replacing the squalene-terminated PEG segments with small, hydrophobic siloxane segments in combinations with the same low molar mass PEI chains (both 0.8 and 2 kDa) [108]. They efficiently bind DNA and form nanometric polyplexes with tight dimensional polydispersity.

**Figure 7:**
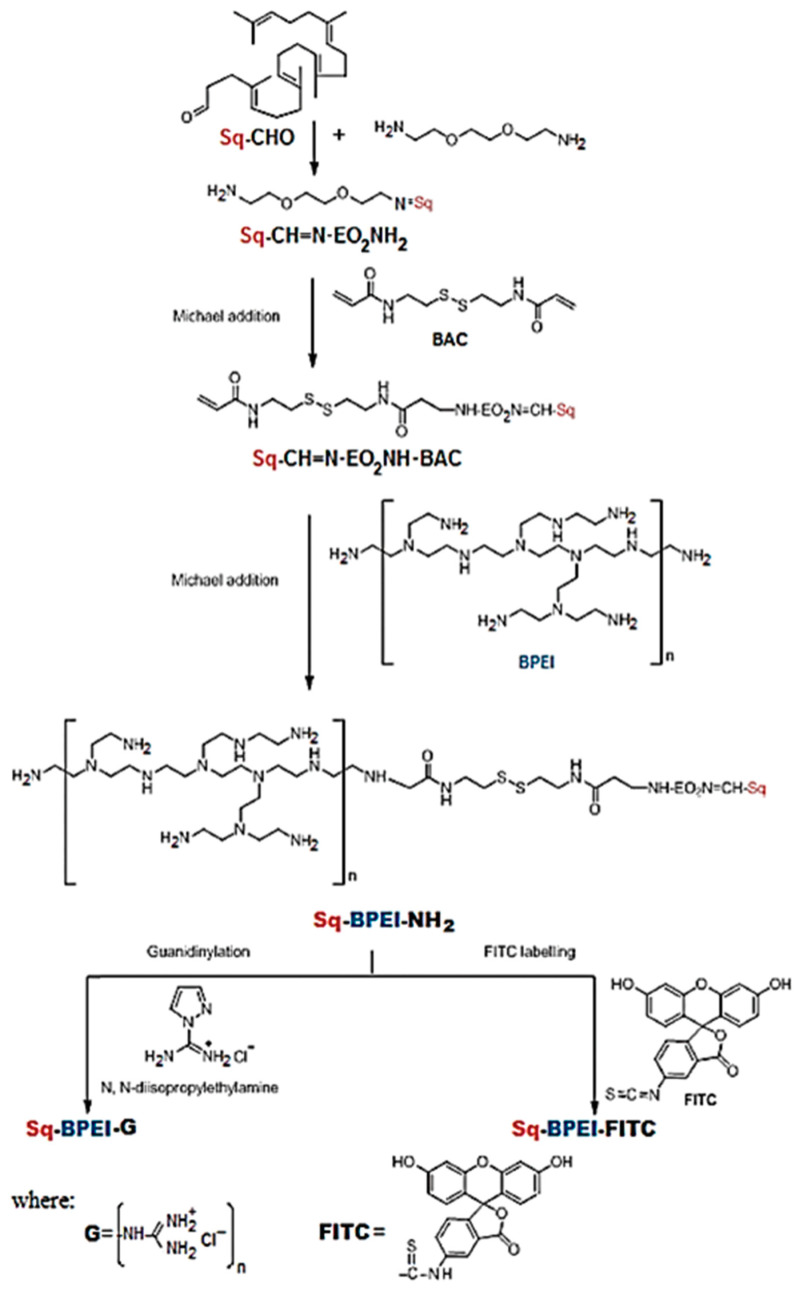
Synthetic pathway towards a water-soluble amphiphilic gene delivery conjugate based on squalene (Sq), branched poly (ethylene imine) (PEI) (bPEI-NH_2_) and a bifunctional aldehyde core (BAC), and its guanidinylated (G) and labeled (FITC) versions. Reproduced from [119], with permission from © 2018 Royal Society of Chemistry.

Satisfying DNA-complexation skills are also attainable when the aforementioned trifunctional aldehyde core is replaced with a bifunctional one (1,1′,2-trisnorsqualenaldehyde) in branched PEI-containing carriers, as can be observed in Figure 7 [119]. The obtained squalene-bPEI amphiphilic conjugate progressively aggregates in an aqueous environment and generates fine, multimolecular micelles of various sizes. The biggest one displayed a diameter below 200 nm, which is beneficial for gene delivery, since polyplex nanoconjugates with dimensions between 10 and 200 nm ensure a long-drawn passage in the vascular system [122].

Hela cells transfection assays show maximum efficiency at N/P ratios of 20 for the squalene-PEI conjugate, and 15 for the guanidinylated derivate, respectively. The outcomes are superior to the ones that are provided by naked bPEI-derived polyplexes with one order of magnitude.

The inclusion of such polyplexes into the biocompatible, macroporous matrix of a hybrid cryogel (based on biopolymers, poly(ε-caprolactone, and PEI-functionalized hydroxyapatite) ensures a non-toxic, constant DNA delivery along 26 days and maximum expression in the 5th day [112,119]. Time-dependent assessment of DNA- and vector-release from the 3D matrix evidenced a modulation of the release kinetics based on electrostatic and hydrophobic interplays involving free carrier in excess, polyplexes and the hybrid cryogel, depending on their specific structural features and the degradation of the microporous network. Static and dynamic experiments point towards the retention of remaining DNA within the matrix along several components (linear or branched PEI, residual biopolymer) that are able to complex the genetic material by their own. Based on the most efficient N/P values, the excess carrier concentration can be further optimized downwards by considering both carrier and hybrid matrix features.

Based on this behavior, it can be considered that the developed platforms enable a tunable release of genetic material and it can be recommended for use as gene-activated matrix systems. These systems merge principles and strategies from gene therapy and tissue engineering to deliver viable alternatives for specific (localized), long-term delivery of genetic material at improved biocompatibility. The pathway unlocks the possibility to bypass some extracellular barriers that are usually hampering any upscaling attempts and imply complex and costly multi-step procedures, and therefore it is highly relevant for clinical applications.

**Figure 8:**
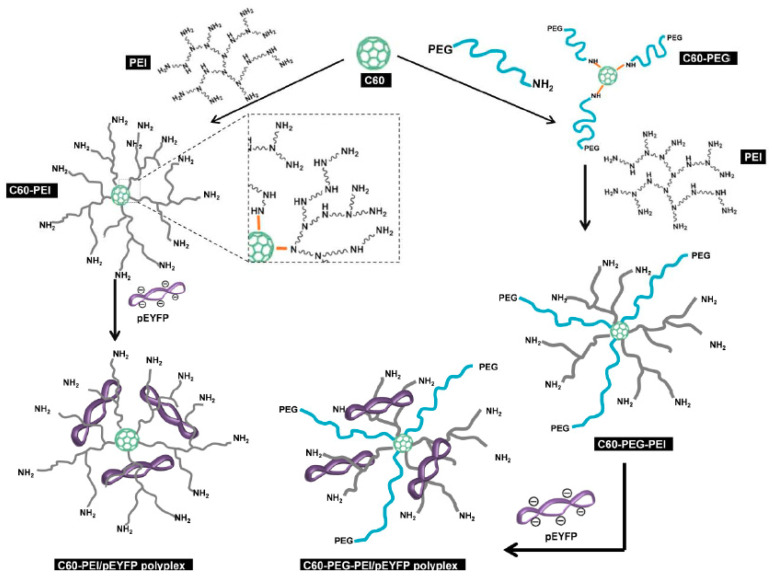
Schematic representation for the formation of fullerene (C60)-based conjugates incorporating relatively short branched PEI linear PEG chains. Reproduced from [114], with permission from © 2018 Royal Society of Chemistry.

Our group has also followed a radical step in the choice of a focal point, by designing and producing fullerene (C60) conjugates with dendrimer-like architectures that efficiently bind linear and plasmidic double stranded DNA and form cytofriendly polyplex-type gene vectors [114]. The non-toxic C60 focal point provides the necessary dimensional and spatial characteristics to bind genetic material, but it suffers from poor solubility. Its conjugation with short bPEI (2 kDa) and the optional addition of PEG chains (also short: 2 kDa) solve this issue and delivers the additional means for proper gene transfection. This approach was used in order to obtain the stable, particulate polyplexes depicted in Figure 8 in a relatively simple and reproducible manner.

The connection of C60 with PEI (1:3.5 molar ratio) or with PEI and PEG (1:2.5:0.9) resulted in soluble, amine-decorated carriers that bind DNA targets (dsDNA or a plasmid carrying a reporter gene, pEYFP in Figure 8) into bioconjugated constructs with diameters between 50 and 200 nm, in direct connection to the type and amount of associated DNA. The polyplexes switch between the anionic and cationic state at an N/P ratio of 5 and reach colloidal steadiness at an N/P value higher than 10, as confirmed by zeta potential measurements and atomic force microscopy, respectively.

The carrier only containing PEI branches showed 25% transfection efficiency of the pEYFP genetic material at N/P values above 20, which is higher than the one obtained for the SuperFect^®^ commercial carrier in the same conditions (15%). PEGylation resulted in conjugates with lower transfection yield (6%), but satisfied its primary goal: low cytotoxic carriers (comparable to the positive control in expressing the EYFP reporter gene in cultured cells) and superior cytocompatibility (200% cell proliferation).

These positive results could be further exploited by including C60 in the aforementioned supramolecular dynamic strategy that was based on libraries of modular carrier frameworks. Moreover, it has already become clinically relevant after its successful use in the delivery of Runx2-shRNA plasmids as to down-regulate the expression of proteins that are involved in the osteodifferentiation of human valvular interstitial cells in diabetic and pro-osteogenic conditions [118].

**Figure 9:**
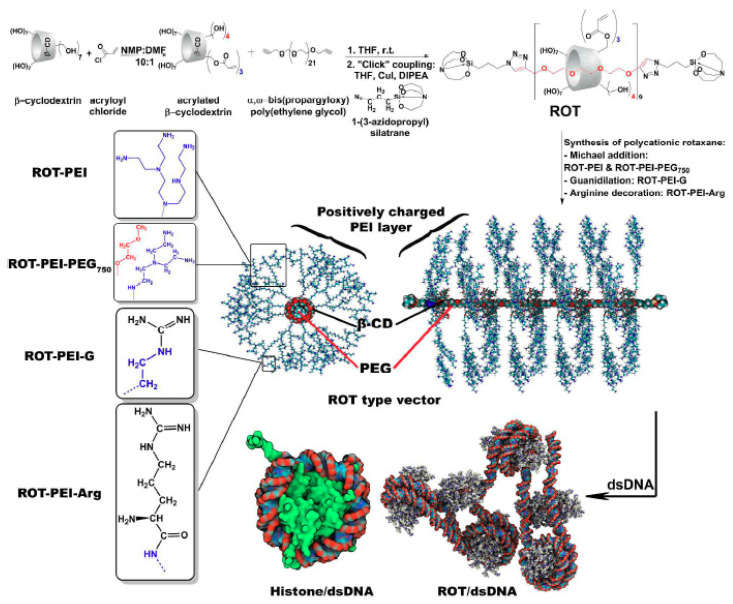
Synthetic pathways towards polyrotaxane-containing carriers and the structural and functional features of cargo-complexes obtained by DNA-binding. Reproduced from [109], with permission from © 2017 Royal Society of Chemistry.

Spectacular results (from the structural blueprint point of view) are accessible when versatile cyclodextrin (CD) entities are used as chain links to develop supramolecular, mechanically interlocked, polyrotaxane assemblies that function as gene delivery vectors through a spatial, cationic network (Figure 9) [109,128].

From the chemical blueprint point of view, the use of CD moieties in the conjugation of amine-decorated polymeric chains (like in the case of PEI) requires their modification with unsaturated structural motifs. The acryloyl chloride synthetic route (conducted in anhydrous and non-oxidative conditions and at a reduced kinetic pace) is one of the most productive pathway in this regard and it also ensures the means for reproducible derivatives (and, as a consequence, reproducible non-viral vectors). The resulting derivatives can be involved afterwards in various synthetic protocols (for example, Michael addition) with the envisaged polymeric backbone [128].

An individual carrier of this type was obtained by employing a flexible PEG axle of definite length (1 kDa) to tread nine functionalized cyclodextrins within two cage-like, silatrane terminal points. Each cyclodextrin moiety was first used to attach three PEI units (2 kDa) in order to generate a giant PEI-like polycation that preserves the DNA-complexation ability but sidesteps the cytotoxicity of high molecular weight PEIs.

The functionality of these cargo-complexes was further multiplied by appending three other structural motifs to the rotaxane side-branches via the pendant PEI branches: a PEI-PEG mixture, guanidine- and arginine-decorated PEI fragments (Figure 9) [109]. While complex and demanding, the accurate design and synthetic strategies deliver reproducible vectors that further function in a reproducible fashion. The resulting framework behaves a positively charged nanoparticle that shares the propensity towards packing large macromolecules of genetic material of the individual, particulate carriers, and leads to compact, tight cargo-complexes.

A combination between the quasi-linear architecture and the cyclodextrin’s mobility enables the nanosized carriers to encircle DNA fragments at their surface and behave as a wrapping/folding/bridging nano-platform. A key feature of the resulting cargo complexes is the ability to chemo-mimic histone octameric complexes and morpho-mimic nucleosomes. In silico molecular modeling, firstly employed to direct the design of the non-viral vector, also corroborated the histone chemo-mimicry of the carrier within the closely-packed, dense, spiral arrangement of DNA, and confirmed its morphological resemblance to nucleosomes [128]. Synergistic cooperation between the carriers’ components unlocks an efficient delivery of DNA into HeLa cells, thus attesting the efficiency of these non-viral vectors.

The interplay between the PEI fragments and cyclodextrin units can be used to tune the organization and composition of such an edifice, while its reactivity towards genetic material and carrier functionality can be tailored by varying the PEI, PEG, and β-CD proportion, and PEI length, respectively.

## 5. Metallic Nanoconjugates for Biomedical Applications

There is one additional chemical tool that can be used in combination with the smart synthetic gadgets presented so far to reach biomedical potential: metals. Metallic nanoparticles with well-defined, controlled size, shape, and dispersity are easily accessible by various preparation methods and they offer many promises for biomedical applications [129,130,131,132,133].

Core-shell magnetic nanoparticles (MNPs) come forward from the wide range of available options, since they favor the engineering of physical features of distinct components for targeted bio-related usage. Three key properties are convenient for biomedical research: the multiple functionalization opportunities, superparamagnetism, and the possibility to specifically guide and accumulate them in a tissue of choice. Therefore, MNPs are already used as imaging agents, drug carriers, and bioactive thermal-based vectors.

One appealing direction in this regard is the employment of MNPs as the carriers of antioxidant agents’ for the inactivation of nitrogen and oxygen reactive species, while assuring minimal side effects [134,135]. Some of our contributions to the field refer to the development of complex, conjugated entities of nanometric dimensions that are capable of transport and release antioxidant agents in a pharmacologically-directed and spatially-guided fashion [136].

Cerium oxide recently evolved as a starred player within inorganic antioxidants, since it provides additional benefits to the radical scavenger activity: radio- and neuro-protection, anti-inflammatory features a.o. Cerium oxide nanoparticles were used as the metallic center of nanoconjugates since they mimic the activity of superoxide dismutase and annihilate reactive oxygen species [137,138].

**Figure 10:**
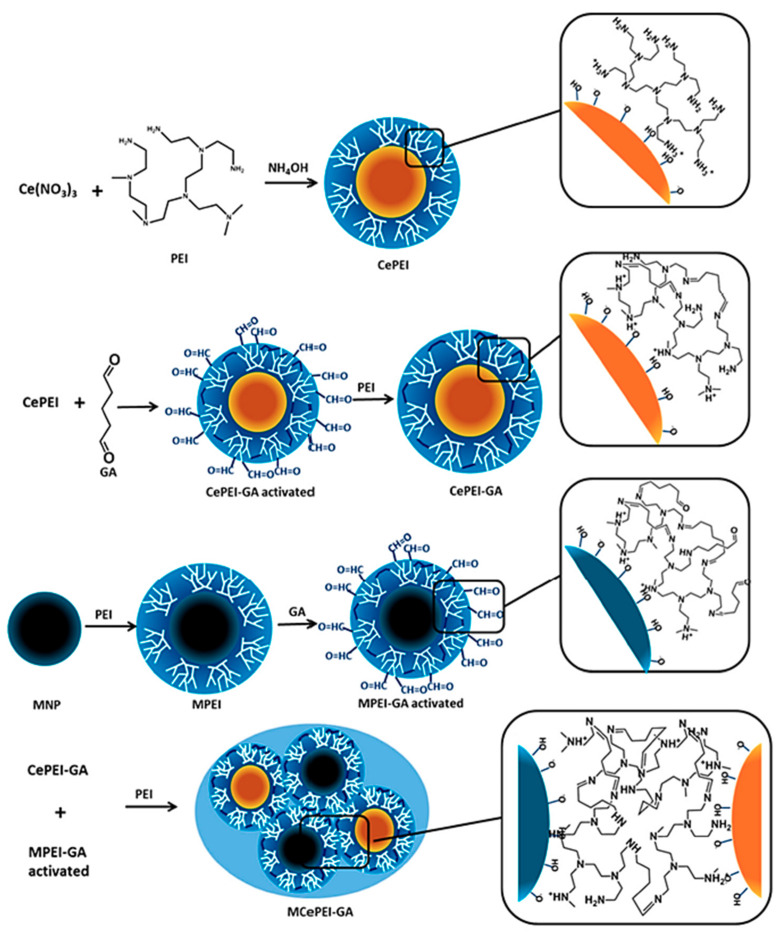
Schematic representation of nanoceria-based nanoconjugates with PEI coatings. Reproduced from [136], with permission from © 2019 MDPI, Basel, under CC BY 4.0.

Therefore, it was also interconnected with magnetite nanoparticles to create the magnetic core of nanosized cargo-complexes, which can be spatially directed in a biological environment via a magnetic field. In both cases, the shell was constructed by coating the metallic nanoparticles with low molar mass (1.8 kDa), branched PEI fragments, in their naked form or after cross-linking and activation with glutaraldehyde (Figure 10). The electrostatic interactions between the cationic polymeric chains and the MNPs with negative zeta-potential stabilize the resulting conjugates and ensure the required steadiness for further physical, chemical, and biological investigations.

The PEI-coated nanoceria conjugates showed a spherical morphology between 5 and 8 nm in diameter, depending on the number of starting components. The two magnetic core-shell complexes display superparamagnetic features and a saturation magnetization within the boundaries of biomedical usage.

They all presented low toxicity and in vitro and in vivo free-radical scavenging features against DPPH (1,1-diphenyl-2-picryl hydrazyl) radicals and ABTS^+•^ (2,2′-azino-bis(3-ethyl-benzthiazoline -6-sulfonic acid) cation radicals, respectively. The oxidative stress reduction was boosted via imine linkages after the activation of the PEI shell and conjugation with magnetic components. The best results were obtained for the magnetic conjugate decorated with activated PEI fragments. When injected in mice, the antioxidant activity of this magnetic conjugate was observed to increase in the order: plasma (9–14%, depending), brain (20%), liver (39%), and spleen (97%). Further investigations are in order to elucidate this mechanism and the synergy between the naturally-occurring antioxidant activity and the nanoconjugate-triggered reduction of oxidative stress.

Nanocarriers prepared by the conjugation of the aforementioned magnetite nanoparticles with small branched PEI fragments (MPEI in Figure 10) were also used to pack a natural free radical scavenger, protocatechuic acid (PCA), or its β-cyclodextrin complex (chemically modified with sulfobutylether to attain anionic units). PCA is seldomly employed as a stabilizer for magnetic particles in aqueous environments when the functionalization is performed through a ligand exchange process. The macrocyclic route became a frequent strategy in the encapsulation of various natural or synthetic antioxidants which suffer from solubility issues. The PEI-based shell increases the stability and it assists the loading, transport, and delivery of the antioxidant, leading to the formation of drug delivery vehicles that fuse the bio-useful traits of the core-shell construct and its load [139].

The antioxidant character of PCA is based on a radical-scavenging ability coming from electron- and hydrogen-donating mechanisms. These antioxidant features were not hampered by its incorporation within the PEI fragments both in its free form and as inserted in the cyclodextrin’s cavity. A higher antioxidant activity was observed in the case of the PEI-based conjugate as compared to the macrocyclic counterpart due to a lower PCA content of the latter (with a final inhibition ratio of 1.28 in favor of the PEI-PCA metallic construct). Release studies presented a steady, three-day discharge and a slower release for the cyclodextrin-containing conjugate, due to the added inclusion level. In addition, in vitro toxicity assays proved the biocompatibility of both bioconjugates.

Therefore, this pathway is a viable solution to deliver unstable, water-insoluble, active principles through biocompatible core-shell conjugates. The mild experimental conditions and relatively facile and reproducible entrapment methods are further benefits in this regard. Moreover, the magnetic guidance and thermic effect of the magnetite component empowers further exploration as a complement to the antioxidative character of these metallic complexes.

## 6. Smart Organic Tools for Biomedical Imaging

The DNA-binding ability of organic structural entities with the help of smart organic tools can also be put to work in other interesting biomedical directions by following the same non-covalent binding tools as most therapeutic species: base pair intercalation, groove- and outside-binding, self-stacking along the biomacromolecule’s backbone a.o.

The addition of a second function to the DNA-binding ability, like a particular emissive behavior, provides the basis to estimate secondary conditions and assess the time-dependent chemical setup of the surrounding biological environment. Therefore, fluorescence imaging evolved in recent years as a robust method to visualize and track biological targets or even processes that occur in living systems [140,141].

This translates into a strong demand for small organic frameworks that are able to bind to certain biomolecules while displaying particular emissive behavior. Any additional feature, like sensitivity or specificity to the transient chemical composition of the biological medium, is welcomed to the field. A wide range of commercial or synthetic organic fluorescent agents is heavily studied and amended to be used in bioimaging and biolabeling. The biggest hurdles to overcome are of analytical (proper photo-physical features to ensure specificity or sensitivity) or biological (unsatisfying solubility, toxicity, poor membrane permeability) nature. From the optical characteristics point of view, one of the most common disadvantages is a narrow Stokes shift (below 25–30 nm) that generates self-quenching and detection errors and, therefore, occludes their applicative promises [142,143,144].

Our group broached the subject by developing a small series of organic dyes with pyridine-indolizinic skeleton and studying their particular, pH-sensitive fluorescence behavior, their DNA-binding affinity, and the possibility to construct supramolecular edifices that are based on them. The indolizine structure proved its usefulness in bioimaging and biosensing, while the planar pyridine-indolizinic framework ensures suitable photo-physical features, especially in terms of Stokes shift values. [145]

**Figure 11:**
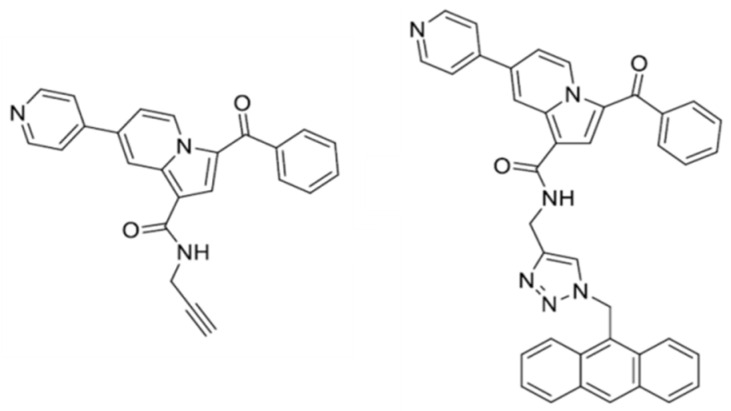
Structure of the pyridyl-indolizines dyes: pendant-propargyl precursor (left) and anthracene-containing dye (right). Adapted from [147], with permission from © 2016 Elsevier.

First, a new 7-(pyridin-4-yl)-substituted indolizine compound with a pendant propargyl unit was developed based on known synthetic procedures [145,146]. This was further subjected to a click reaction with an anthracene azide derivative to prepare a pyridyl-indolizine dye that anchors an anthracene moiety (Figure 11) [147]. The rigid aromatic anthracene functionality was chosen due to its well-established electro-optical features and the propensity towards aromatic interactions that could further promote structural interplays within the helical DNA backbone [148,149,150]. Anthracene derivatives (some of them being used as chemotherapeutic agents) strongly bind to DNA in biological environments based on a intercalating and groove-binding mechanism

Both of the derivatives acted as yellow fluorophores and proved distinct absorption and emission features in direct correlation with the pH value and dye structure (especially the protonation of the pyridine unit). High Stokes shift values were calculated for the optical properties in buffer solutions and they proved to be comparable or even superior to the ones that were reported for other indolizine derivatives in organic media.

The addition of DNA at physiological pH modified the absorption and emission spectra of both dyes, indicating their performance as nucleic acid binders, which was further confirmed by gel electrophoresis experiments. Moreover, the anthracene derivative shows weak fluorescence intensity in aqueous media due to an aggregation-based quenching process that is instantly suspended in the company of the biological macromolecule, as evidenced by the sharp fluorescence increase.

Molecular docking studies correlated with the photo-physical experimental information indicated that the anthracene-based derivative displays a higher affinity towards DNA as its propargylic counterpart, as revealed by the lower binding energy and complex dissociation constant values. These results are the starting points of consecutive in-depth studies in order to confirm the DNA-binding mechanism and address DNA photocleavage procedures based on the anticancer prospects of the anthracene-derived system. However, any research on the matter should be preceded by the optimization of the dyes’ structure to unlock enhanced solubility and non-toxicity.

**Figure 12:**
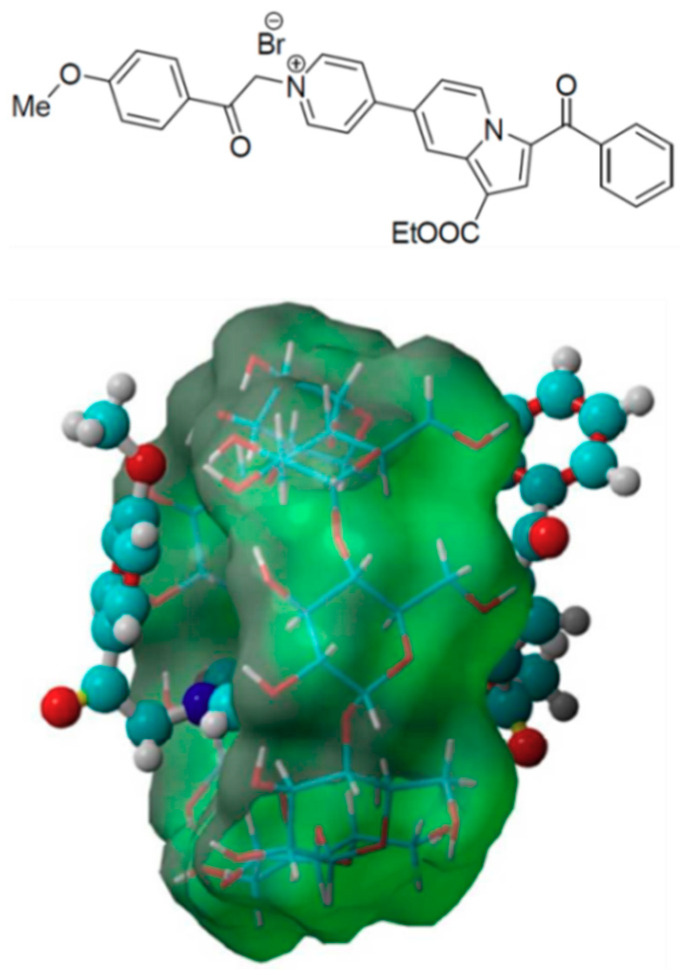
Structure of the pyridinium-indolizine dye and molecular docking model of its 1:1 inclusion complex with β-cyclodextrin. Adapted from [154], with permission from © 2017 Royal Society of Chemistry.

Based on the improvements obtained in the case of commercial fluorescent dyes used as imaging agents, one direction of research is the use of supramolecular host-guest assemblies involving cyclodextrins [151,152,153]. For example, another indolizine derivative containing a pyridinium salt fragment, which suffers from high toxicity and reduced solubility issues was introduced into inclusion complexes with β-cyclodextrin in 1:1 and 1:2 ratios (Figure 12).

The pyridinium salt is prone to base-triggered, reversible formation of nitrogen ylides. This feature imparts a pH-sensitive fluorescence emission, which is not severely hampered by the incorporation in β-cyclodextrin assemblies [154].

The resulting assemblies annulled the dye’s toxicity and, more interesting, showed cellular permeability and selective accumulation within cell acidic organelles (lysosomes or mitochondria). To our best knowledge, this is the first corroboration of a cyclodextrin-triggered toxicity mitigation of a fluorescent dye coupled with a cell staining validation. A 48-h study regarding emissive properties in biological media evidenced a long-lived, constant intracellular fluorescent behavior, superior to commercially available organelles markers.

The excellent intracellular dye stability qualifies the inclusion complexes as viable candidates for acidic organelles labeling or staining and it suggests an expansion towards the assessment of lysosomal morphology and trafficking in intact cells. Moreover, they imply that the host-guest pathway should be expanded to other fluorescent agents with poor solubility and limiting toxicity, as to widen the current compact collection of fluorescent agents for cells staining or labelling.

## 7. Conclusions and Future Directions

This minireview highlights some particular developments obtained in the last five years by our group on the topic of stimuli-sensitive molecular tools for biomedical applications. The design and mechanistic details are provided concerning the smart synthetic pathways that are employed to prepare supra- and macro-molecular architectures with specific responses to external stimuli. Five major themes are approached: (i) temperature- and pH-responsive systems for controlled drug delivery; (ii) glycodynameric hydrogels for drug delivery; (iii) polymeric non-viral vectors for gene delivery; (iv) metallic nanoconjugates for biomedical applications; and, (v) smart organic tools for biomedical imaging.

The dynamic transformation of these materials under external triggers comes close to the much sought after biological intelligence that inspired the smart polymers area. The progress attained so far promises to settle several issues in the field: safe and specific delivery of targeted bioactive agents, enhanced sensitivity and response, improved stability and solubility, superior exploration of supramolecular assemblies, biomedical possibilities a.o. Moreover, it provides some starting points in the quest for smart solutions for the futuristic challenges of the ever-demanding biomedical research.

It can be considered that the temperature- and pH-responsive systems for controlled drug delivery or smart dyes for biomedical imaging reached the necessary maturity to go to the next step, in terms of upscaling, testing level, or even market entry. Meanwhile, the true potential of glycodynamers, non-viral vectors, and metallic conjugates is still emerging and there is quite a long road to be covered before their final use. There are many synthetic pathways to be enhanced, mechanistic insights to be discovered, polymeric architectures to be constructed, and novel features to be explored.

A broader extension of the dynamic imine-based strategy that is based on the use of new, bio-inspired monoaldehydes or other biomacromolecules of polysaccharide nature is expected in order to pave the way for new applicative promises, especially in the drug delivery area. Moreover, morphology control and thixotropy exploration could further boost the research in this direction and generate interesting results.

Satisfying DNA-complexation skills are attainable via the supramolecular architectures presented herein. The self-adjustment strategy and library-based pathway proved their highly productive and further development of synthetic platforms based on other focal points and/or macromolecular building blocks is expected in the near future. However, the novel non-viral vectors are forced to satisfy new, higher standards in terms of functionality and efficiency to get closer to clinical trials. Moreover, the clinical relevance of the gene-activated matrix systems strategy empowers the quest for new components of such combined systems.

Core-shell magnetic nanoconjugates represent an intense research subject. The work performed so far delivered biocompatible, stable, and very active constructs. Further studies are required in order to unveil mechanistic insights and build some connections between the innate antioxidant activity and the oxidative stress reduction determined by the nanoconjugate.

DNA-binding, smart organic frameworks seem to have the smallest hurdles to pass towards application (especially in bioimaging and biolabeling). While the required stability, specificity, and sensitivity were already attained, there is still some non-trivial work to be performed in order to ensure proper non-toxicity and membrane permeability, and the cyclodextrin-based blueprint looks promising in this regard.

There is one additional tool that brings key benefits in all of these research topics: in silico chemistry. As scarcely mentioned throughout the review, theoretical molecular dynamics simulations, logistic models, molecular docking studies, and design of experiments have high impact in all stages of research, from the initial design to the fine modulation of resulting systems. Data-driven models enable the foundation of complex functional relationships between various levels of design and experimental factors and the final features or efficacy (response) of the obtained materials.
